# A clinical approach to tubulopathies in children and young adults

**DOI:** 10.1007/s00467-022-05606-1

**Published:** 2022-05-18

**Authors:** Rachael Kermond, Andrew Mallett, Hugh McCarthy

**Affiliations:** 1 Department of Renal Medicine, Sydney Children’s Hospital Network, Sydney, NSW Australia; 2 Department of Renal Medicine, Townsville University Hospital, Douglas, QLD Australia; 3 College of Medicine and Dentistry, James Cook University, Douglas, QLD Australia; 4 Institute for Molecular Bioscience & Faculty of Medicine, The University of Queensland, Brisbane, QLD Australia; 5 Faculty of Medicine and Health, The University of Sydney, Sydney, NSW Australia; 6 Centre for Kidney Research, The Children’s Hospital at Westmead, Sydney, New South Wales Australia

**Keywords:** Tubulopathy, Salt-wasting, Magnesium, Hypokalaemia, Genetics, Nephrocalcinosis, Rare disease

## Abstract

Kidney tubules are responsible for the preservation of fluid, electrolyte and acid-base homeostasis via passive and active mechanisms. These physiological processes can be disrupted by inherited or acquired aetiologies. The net result is a tubulopathy. It is important to make a prompt and accurate diagnosis of tubulopathies in children and young adults. This allows timely and appropriate management, including disease-specific therapies, and avoids complications such as growth failure. Tubulopathies can present with a variety of non-specific clinical features which can be diagnostically challenging. In this review, we build from this common anatomical and physiological understanding to present a tangible appreciation of tubulopathies as they are likely to be clinically encountered among affected children and young adults.

## Introduction

Fluid, electrolyte and acid-base homeostasis are imperative for the preservation of life. This is accomplished by adjustments in glomerular filtration and tubular reabsorption of solutes and fluids in response to fluctuations in dietary intake and metabolic processes [1, 2]. As a complex, closely regulated and interdependent series of physiological processes, absolute or relative dysfunction is of critical relevance to the health and development of disease.

**Fig. 1:**
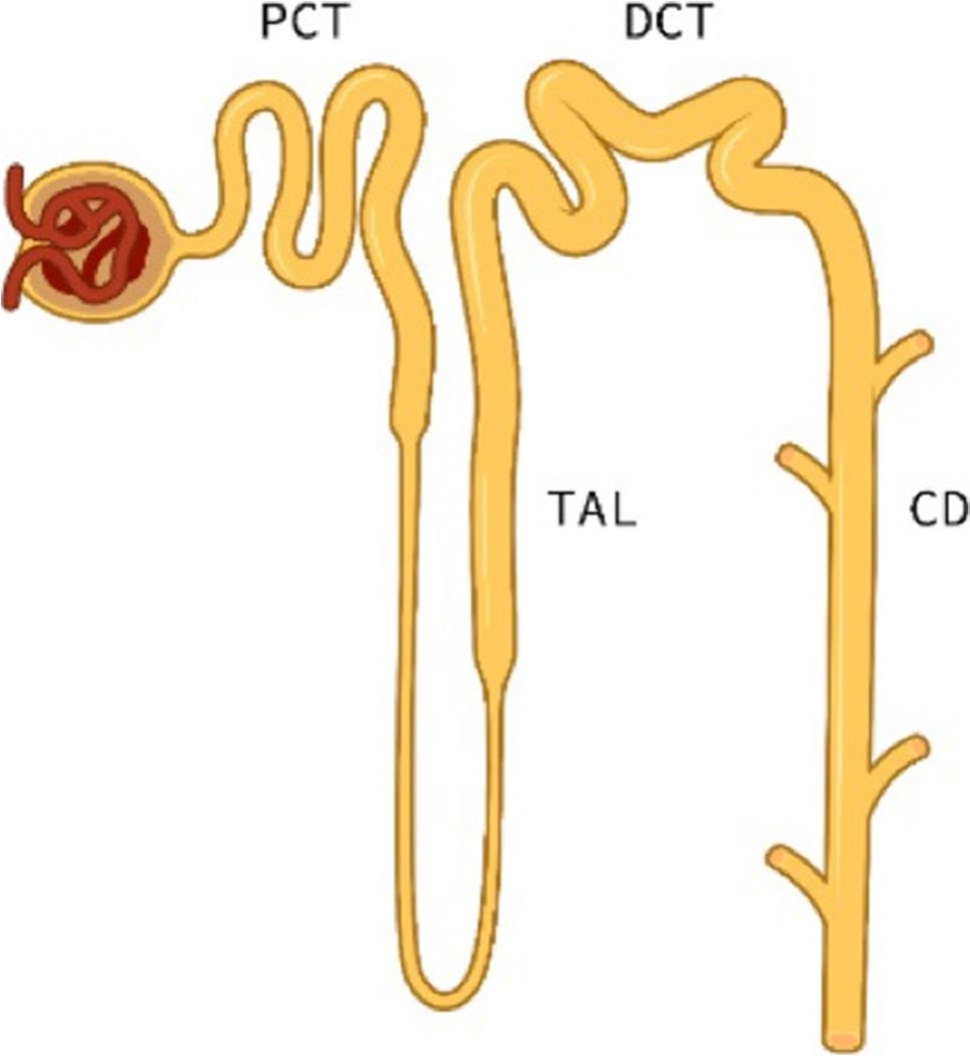
The nephron with associated segments

Kidney tubules are typically divided into four broad segments based on anatomical and functional characteristics (Fig. 1) [2]. The proximal convoluted tubule (PCT) is responsible for the reabsorption of the majority of water and solutes including amino acids, low-molecular-weight proteins (LMWPs) and glucose. The PCT has a significant energy requirement and is vulnerable to conditions that result in an impaired energy supply such as inborn errors of metabolism [3–5].

Following the PCT is the thick ascending loop of Henle (TAL) [2]. The TAL provides the urinary concentrating mechanism enabling tubular excretion of solutes with minimal water loss [6]. It reabsorbs up to 30% of filtered sodium via the Na-K-2CL cotransporter (NKCC2) and contributes to calcium and magnesium homeostasis via paracellular mechanisms [7].

The distal tubule is composed of the distal convoluted tubule (DCT) and the collecting duct (CD). Sodium and water reabsorption is highly variable in these segments secondary to the mineralocorticoid (aldosterone)-responsive principal and intercalated cells [8, 9]. The principal cells increase sodium and therefore water reabsorption via aldosterone-mediated activation of the epithelial sodium channel (ENaC) [10]. In times of potassium excess, the intercalated cells facilitate potassium secretion via the same mechanism. Aldosterone is released, and ENaC stimulated, resulting in an electrochemical gradient which promotes potassium secretion via the ROMK channel [9, 11]. Intercalated cells are also responsible for acid-base homeostasis with the excretion of hydrogen and reabsorption of filtered bicarbonate [9].

Unique to the DCT, the thiazide-sensitive NaCl cotransporter reabsorbs 5–10% of filtered sodium [8]. The DCT also contributes to calcium and magnesium homeostasis via transcellular mechanisms and is responsible for the secretion of potassium via both voltage and flow-dependent processes [2, 8].

Finally, the principal cells within the CD also facilitate water reabsorption via the water channel aquaporin-2 (AQP2) which is stimulated by the antidiuretic hormone (ADH) [12].

The dysfunction of any of these tubular mechanisms results in a “tubulopathy”.

## Clinical presentation

The clinical presentation of tubular dysfunction in children and young adults is as equally varied as it is non-specific. Prominent features include polyuria, polydipsia, irritability, growth failure, nephrocalcinosis and blood pressure anomalies.

It is important to elicit a history of polyuria and or polydipsia as these reflect a concentrating defect. This can be compounded by an osmotic diuresis secondary to increased solute delivery to the distal tubule. The body compensates with the release of ADH and aldosterone (if these mechanisms remain intact) and increased thirst. The loss of water and solutes, however, is constant, and despite polydipsia, children are often chronically or intermittently dehydrated resulting in irritability.

Growth failure is a common presenting feature and ongoing management issue. The magnitude of the growth deficit is multifactorial and dependent on the severity of the underlying tubulopathy. Chronic acidosis results in protein catabolism and growth hormone deficiency/resistance and has a direct effect on the epiphyseal growth plate [13]. Chronic hypokalaemia, a common feature of selected tubulopathies, has also been associated with growth hormone deficiency and resistance [13, 14]. Phosphate and calcium wasting, and subsequent rickets secondary to kidney disease, have an obvious impact on growth. Finally, polyuria and polydipsia result in significant fluid intake impacting the ability of (particularly young) children to meet their caloric requirements to facilitate growth.

A differentiating feature of many tubulopathies is hypercalciuria and nephrocalcinosis, additionally manifesting as nephrolithiasis. Hypercalciuria can occur secondary to increased absorption of calcium from the gut (e.g. in vitamin D toxicity), increased release of calcium from bone (in the setting of acidosis) and reduced reabsorption of calcium in the tubule as is the case for many tubulopathies [15].

Blood pressure effects vary. In tubulopathies that result in water or salt retention hypertension is observed and often marked (e.g. Liddle syndrome). In those with salt and water wasting, the net effect is hypo- or normotension (e.g. Bartter Syndrome). It is important to note that hypertension can still be present in salt-losing tubulopathies, for example secondary hypertension noted in adults with Gitelman Syndrome [16].

Other potential extra-kidney manifestations including sensorineural hearing loss, ophthalmologic involvement and developmental delay could guide the diagnosis in the direction of specific tubulopathies.

## Investigation

When faced with a potential tubulopathy, it is important to have a diagnostic tool kit to differentiate these conditions. These include urine and blood tests, imaging and, more recently, genetic assessment (Table 1).

**Table 1:** Initial investigations (biochemical profile including Na^+^, K^+^, Cl^*−*^, HC0_3_^*−*^, urea, creatinine, Ca^*2*+^, Mg^*2*+^, PO_4_^3*−*^)

Bloods	Urine	Imaging
Venous blood gas Biochemical profile +/− Osmolality Renin/aldosterone	Urinary dipstick for glucose Urine microscopy Urine protein:creatinine ratio Urine calcium:creatinine ratio +/− Urine B2 microglobulin Urine osmolality Urine metabolic screen	Kidney ultrasound

Serum evaluation should include a venous gas to assess acid-base status, full biochemical profile including electrolytes, urea and creatinine, calcium, magnesium, phosphate and uric acid to determine the salt-wasting profile. Renin and aldosterone are important in those who present with potassium abnormalities (hypo/hyperkalaemia) with or without hypertension.

Urine should be analysed with urine dipstick (for glucosuria), protein:creatinine ratio and calcium:creatinine ratio. Additional investigations for suspected proximal tubulopathy include beta-2 microglobulin (or equivalent tubular protein) and urine amino acid profile or metabolic screen. Beta-2 microglobulin is a LMWP freely filtered by the glomerulus and reabsorbed by the tubules; therefore, abnormally elevated levels in the urine can be suggestive of tubular dysfunction.

If initial testing is suggestive of a tubulopathy, paired urine and serum samples should be obtained to enable the calculation of fractional excretion of sodium (FeNA) and magnesium (FeMg) in addition to the trans tubular potassium gradient (TTKG) and tubular maximum phosphate reabsorption per glomerular filtration rate (TmP/GFR) (Table 2). These fractional excretions can provide an insight into the electrolyte handling of the kidneys and evidence of the underlying pathology.

**Table 2:** Assessment of tubular handling of salts [55, 56]

Fractional excretions			
	Formula	Normal value	Interpretation
FeNa	$=\frac{Na\ (urine)x\ Creatinine\ (serum)}{Na\ (serum)x\ creatinine\ (urine)}x100$	FeNa <1% *(with normal salt load and normal GFR)*	If >1% suggests: – Kidney salt wasting – Appropriate naturesis in the context of salt load
FeMg	$=\frac{Mg\ (urine)x\ Creatinine\ (serum)}{Mg\ (serum)x\ creatinine\ (urine)x\ 0.7\ }x100$	FeMg < 4%	> 4% suggests – Kidney wasting magnesium in setting of hypogmagnesaemia
TTKG	$=\frac{K\ (urine)x\ Osmlality\ (serum)}{K\ (serum)x\ Osmolality\ (urine)}x100$	TTKG 4–6% *(Interpretation dependent on kalaemic state)*	In hypokalaemic states – <2% suggests appropriate kidney handling – >4% suggests kidney losses
TmP/GFR	$= PO4\ (serum)\left[\mathrm{PO}4\ \left(\mathrm{urine}\right)x\ \mathrm{Creatinine}\frac{\mathrm{serum}}{\ \mathrm{urine}}\right]$	Varies with age	< lower limit of range – Kidney phosphate wasting Ranges: Birth: 1.43–3.43 mmol/L 3 mths: 1.48–3.30 mmol/L 6 mths: 1.15–2.60 mmol/L 2–15 years 1.15–2.44 mmol/L

A kidney ultrasound will detect nephrocalcinosis and/or nephrolithiasis, hydronephrosis in those with polyuria and congenital anomalies of the kidney and urinary tract (CAKUT) which can be associated with tubular pathologies such as *HNF1B-*associated disease.

Finally, a genetic assessment should be considered where feasible. There are now over 50 disease genes implicated in tubulopathies, with some disorders having very specific phenotypes where others exhibit crossover or phenocopy phenomena [1]. The diagnostic yield for genetic testing in tubulopathies is much greater than many other conditions with up to 50% of paediatric or young adult patients having an identifiable genetic diagnosis [2, 17]. Where possible, genetic testing can be utilised for confirmation of diagnosis, guiding disease-specific therapy, providing prognostication, identification of at-risk relatives and antenatal counselling for future pregnancies [1]. Nonetheless, the availability and financial viability of these assessments vary from centre to centre and they are not imperative for diagnosis though can often provide personalised clinical utility.

## Biochemical presentations

Given the non-specific clinical presentations of tubulopathies, it is essential to utilise observed biochemical changes to localise which tubular segment may be implicated. These changes reflect a disruption of normal tubular physiology and their distinctive biochemical patterns are reflective of the disease process [1]. Such biochemical patterns include hypokalaemic or hyperkalaemic metabolic acidosis and hypokalaemic metabolic alkalosis. Additional factors such as family history and syndromic features can assist in determining the underlying diagnosis.

### Hypokalaemic metabolic acidosis

Hypokalaemic metabolic acidosis is a typical feature of both proximal tubular bicarbonate wasting and impaired acid secretion in the distal tubule [3].

### *Proximal tubular bicarbonate wasting*

In the PCT, impaired co-transport of sodium and bicarbonate results in acidosis and volume depletion. This stimulates renin-angiotensin-aldosterone system (RAAS) activation leading to the exchange of potassium ions for sodium in the distal tubule and ultimately hypokalaemia.

Hypokalaemic metabolic acidosis when secondary to proximal tubular dysfunction is most commonly associated with the wasting of all solutes reabsorbed in PCT. This **generalised proximal tubulopathy (GPT)** may also be referred to as **Renal Fanconi Syndrome** [4]. In a GPT, sodium, potassium, calcium, phosphate, uric acid, bicarbonate, glucose, amino acid and LMWP wasting occurs. The net result is hypokalaemic metabolic acidosis with hypo/normotension (due to salt and water wasting), hypophosphataemia with subsequent rickets, hypercalciuria with nephrocalcinosis, glucosuria, aminoaciduria, and LMW proteinuria. Calculating the FeNa can be useful in this setting of reduced extracellular volume as it is expected FeNa would be < 1%; however, in GPT, FeNa is often inappropriately elevated (> 1%) due to salt wasting. It is important to be mindful that FeNa is also affected by salt intake (and therefore not a diagnostic tool of value in breastfeeding infants without free access to salt) and GFR and therefore not always helpful. TTKG will be elevated and TmP/GFR inappropriately low [4]. Clinically these children generally present in the first year of life with failure to thrive, polyuria/polydipsia, irritability, vomiting and growth failure with evidence of rickets [4].

**GPT** has an extensive list of genetic and acquired causes. The genetic causes can be categorised according to the mechanism of dysfunction including accumulation of toxic metabolites (e.g. cystinosis, galactosaemia, Wilson’s disease), impaired energy production (mitochondrial cytopathies) or disruption of intracellular messaging (Lowe syndrome, Dent disease) [4]. These multisystem diseases are associated with extra-kidney manifestations such as ophthalmological involvement, developmental delay and/or hepatomegaly. There are four genes identified to date which result in GPT with a kidney-limited phenotype. Three are autosomal dominant (AD) (*GATM*, *EHHADH*, *HNF4A*) and one autosomal recessively (AR) inherited (*SLC34A1*). Acquired causes of GPT include medications (aminoglycosides, ifosfamide), toxins (heavy metal poisoning) and kidney injury (acute interstitial nephritis, recovering acute tubular necrosis) [2].

**Lowe syndrome **(Oculocerebral syndrome) and **Dent disease (types 1 and 2)** present with GPT with unique characteristics. Both are X-linked recessive disorders associated with an incomplete proximal tubulopathy predominated by LMW proteinuria, marked hypercalciuria with significant nephrocalcinosis and progressive CKD. Importantly, they rarely have glucosuria [18]. Lowe syndrome and Dent-2 disease are both caused by variants in *OCRL* which encodes for an enzyme important in the PCT endolysosomal pathway [18]. Lowe syndrome is associated with devastating extra-kidney manifestations including cataracts, glaucoma, visual impairment, intellectual impairment, behavioural regression and seizures [19]. Interestingly, extra-kidney manifestations in Dent-2 disease are less common. It is hypothesised that these conditions represent variable phenotypic expression of the *OCRL* gene product. Dent-1 disease is caused by variants in *CLCN5*, accounts for 60% of patients with Dent disease and rarely has extra-kidney manifestations [18].

A rare cause of hypokalaemic metabolic acidosis secondary to bicarbonate wasting that is not associated with GPT dysfunction is **isolated proximal renal tubular acidosis (RTA)** also referred to as **type 2 RTA**. This condition is caused by *SLC4A4* variants (encodes for basolateral sodium bicarbonate exchanger) and is associated with eye abnormalities such as cataracts, glaucoma and band keratopathy [20]. Differentiating features of this condition include the lack of amino acid wasting and the absence of nephrocalcinosis. Nephrocalcinosis is thought to be prevented in this condition due to significant citraturia which prevents calcium precipitation and stone formation [21].

### *Impaired hydrogen excretion in the distal tubule*

Hypokalaemic metabolic acidosis secondary to distal tubular dysfunction is due to failure of the intercalated cells to secrete protons. To counter this, sodium is reabsorbed in the CD in exchange for either protons or potassium. If there is no capability to excrete protons, potassium will be preferentially excreted resulting in hypokalaemia. The chronically acidotic state results in calcium release from bones which contributes to hypercalciuria and nephrocalcinosis [20].

**Distal RTA**, also referred to as **type 1 RTA** is an example of this. There are five genes associated with this phenotype, three of which are also associated with sensorineural hearing loss (*ATP6V1B1*, *ATP6V0A4*, *FOXI1*) and two which are not (*SLC4A1*, *WDR72*). There are also several acquired causes of distal RTA including CKD, lupus nephritis or medication related (e.g. amphotericin B) [20–22].

**Fig. 2:**
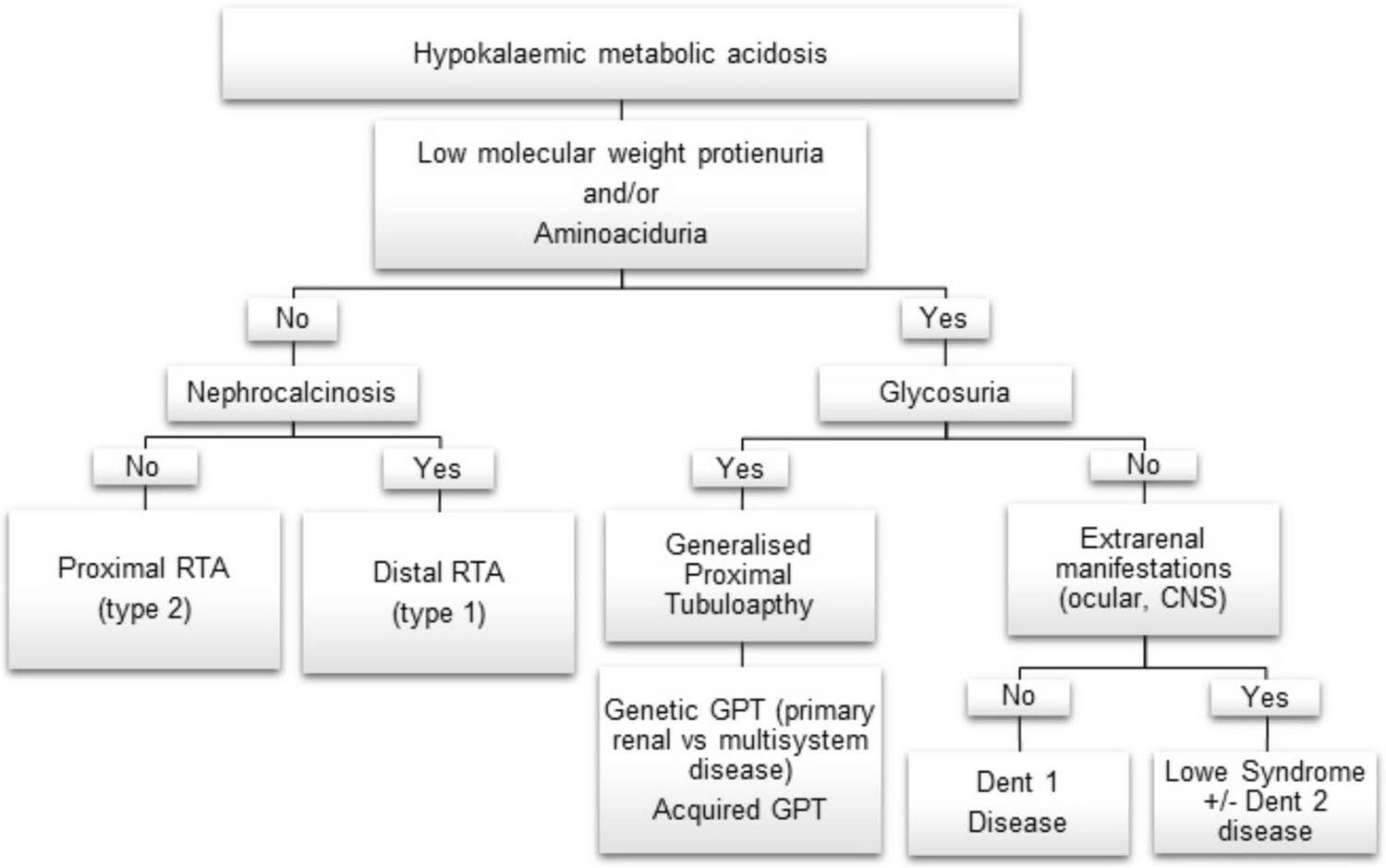
Diagnostic flow chart for hypokalaemic metabolic acidosis

The clinical presentation of distal RTA again includes irritability and vomiting, poor lineal growth secondary to an acidotic state, rickets and sensorineural hearing loss. Biochemically these patients demonstrate hypokalaemic metabolic acidosis with hypercalciuria, nephrocalcinosis with no glucosuria, aminoacidouria or LMW proteinuria to suggest proximal tubule involvement (Fig. 2).

### Hypokalaemic metabolic alkalosis

Hypokalaemic hypochloraemic metabolic alkalosis is the hallmark of enhanced sodium reabsorption in the CD. Sodium reabsorption here is mediated by aldosterone release in response to intravascular fluid depletion. Aldosterone increases the expression of sodium channels and sodium-potassium ATPase within the tubular cell enhancing sodium reabsorption (and therefore water) at the expense of potassium. Potassium depletion is detected and potassium exchanged for hydrogen (via potassium hydrogen ATPase) in the intercalated cells resulting in an alkalotic state [3, 11, 23].

Important differentials for this state include pyloric stenosis, congenital chloride diarrhoea, cystic fibrosis and chronic laxative or diuretic use [23].

### *Hypokalaemic metabolic alkalosis with hypotension/normotension*

The two most common kidney causes are **Bartter Syndrome** and **Gitelman syndrome**. These are salt wasting disorders secondary to variants in genes encoding the sodium co-transports within the TAL and DCT respectively. Other differentials include EAST syndrome, HNF1B-associated tubulopathy and more recently described variants in *CLDN10*, *RRAGD* and mitochondrial DNA [24–27].

**Fig. 3:**
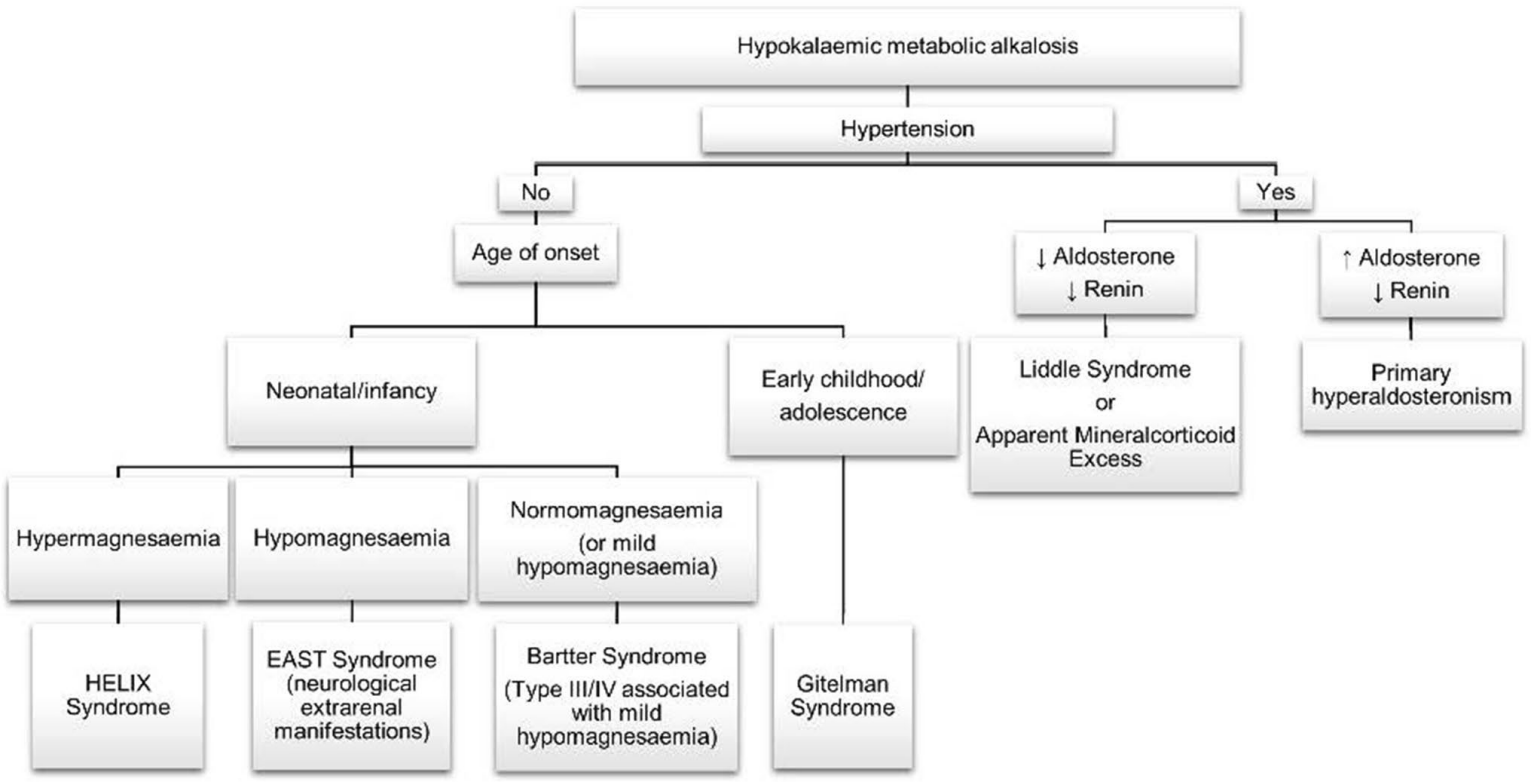
Diagnostic flow chart for hypokalaemic metabolic alkalosis

**Fig. 4:**
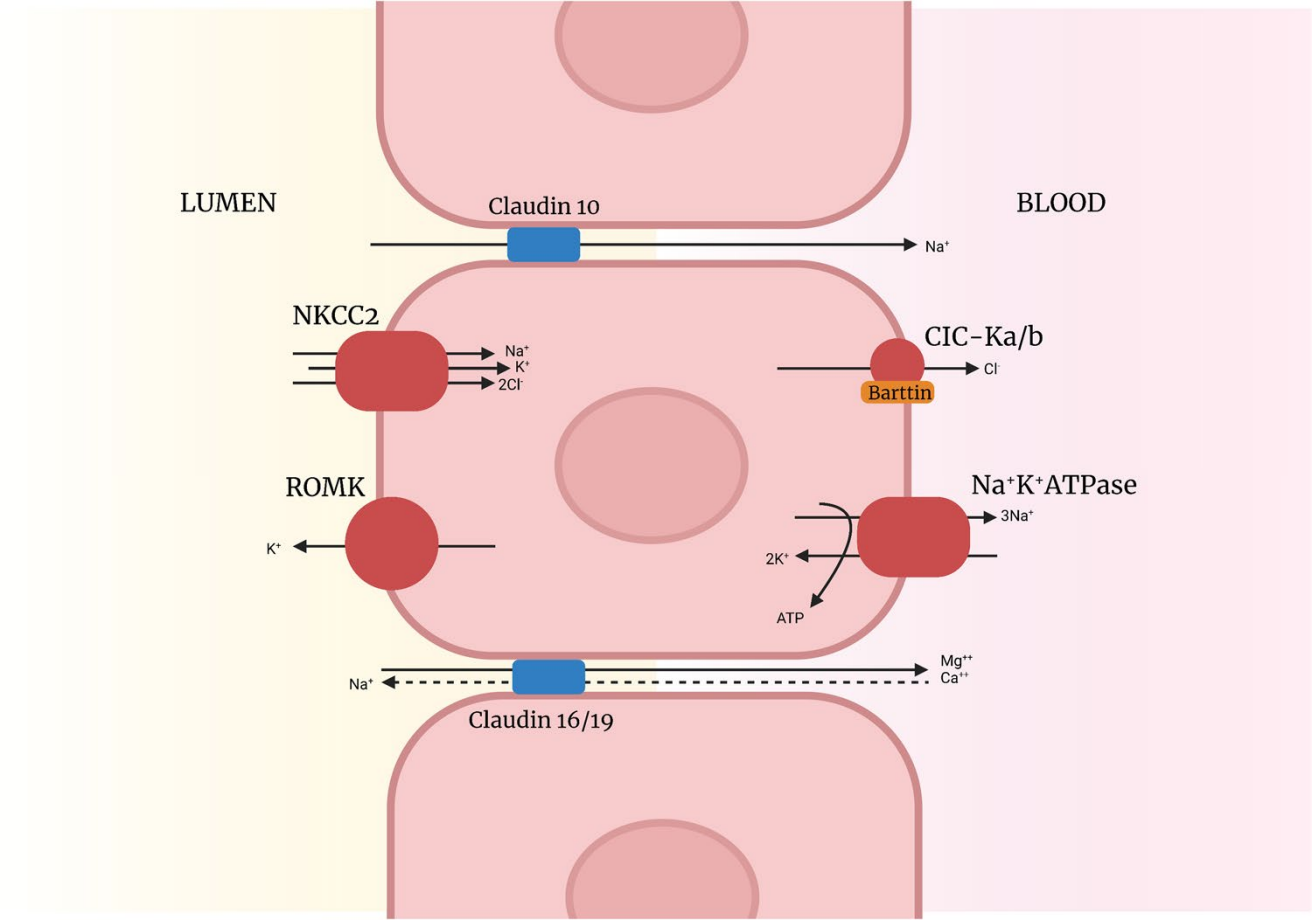
Thick ascending limb of the Loop of Henle with the NKCC2, ROMK, Barrtin subunit and CIC-Ka/b implicated Bartter syndrome. Also demonstrates paracellular reabsorption of sodium, magnesium and calcium via tight junction transmembrane proteins claudin 10, 16 and 19

**Bartter syndrome (BS)** is further classified into types I–V based on underlying genetic diagnosis (Table 3, Fig. 3). All variants affect sodium transport within the TAL resulting in salt wasting with subsequent compensatory hyperaldosteronism (Fig. 4). Clinically these patients can present antenatally with polyhydramnios (due to polyuria) or in infancy/early childhood with polyuria, polydipsia, failure to thrive and complications of chronic hypokalaemia including rhabdomyolysis and cardiac arrhythmias. Interestingly, type V BS presents antenatally and is transient, resolving within the first 3 months of life; the remainder persist into adult life and require prompt diagnosis in the neonatal period or early childhood due to the high risk of mortality [25].

**Table 3:** Differentiating hypokalaemic metabolic alkalosis with normo/hypotension

Hypokalaemic metabolic alkalosis				
Disorder	Gene	Protein	Inheritance	Differentiating features
BS Type I	*SLC12A1*	NKCC2 Co-transporter	AR	Antenatal or neonatal onset
				Nephrocalcinosis
BS Type II	*KCNJ1*	ROMK potassium channel	AR	Antenatal or neonatal onset
				Nephrocalcinosis
BS Type III	*CLCNKB*	CIC-Kb	AR	Childhood onset
		chloride channel		Hypomagnesaemia, can have normocalciuria and phenocopy Gitelman Syndrome
BS Type IVa	*BSND*	Barttin	AR	Neonatal onset
		subunit		Hypomagnesaemia, sensorineural deafness
BS Type IVb	*CLCNKA*	CIC-Ka Chloride Channel	AR	Neonatal onset
				Sensorineural deafness
BS Type V	*MAGED2*	MAGE-D2	XR	Antenatal or neonatal onset
				Transient
Gitelman Syndrome	*SLC12A3*	NCC co-transporter	AR	Childhood onset
				Hypomagnesaemia, hypocalciuria
Gitelman-like syndrome	*MT-TF*	NCC co-transporter	Mt	Childhood onset
	*MT-TI*			Hyomagnasaemia, hypocalciuria
Gitelman-like syndrome	*RRAGD*	Rag GTPase D	AD	Hyomagnasaemia, nephrocalcinosis
				Dilated cardiomyopathy
East Syndrome	*KCNJ10*	Kir4.1	AR	Infancy
				CNS involvement
Helix Syndrome	*CLDN10*	Claudin-10b complex	AR	Childhood
				Hypermagnesaemia
				Exocrine gland dysfunction

**Gitelman syndrome (GS)** is more common than BS, occurring in 1:25,000 births [2]. It results from salt and chloride wasting with secondary hyperaldosteronism due to variants in the gene encoding the NCC in the DCT *(SLC12A3)*. Children with this condition generally present later in life (early childhood or adolescence) with polyuria, polydipsia and hypotension. Symptomatic hypomagnesaemia with muscle cramps, tetany, chondrocalcinosis is an important differentiating feature of GS [2, 28, 29].

**Fig. 5:**
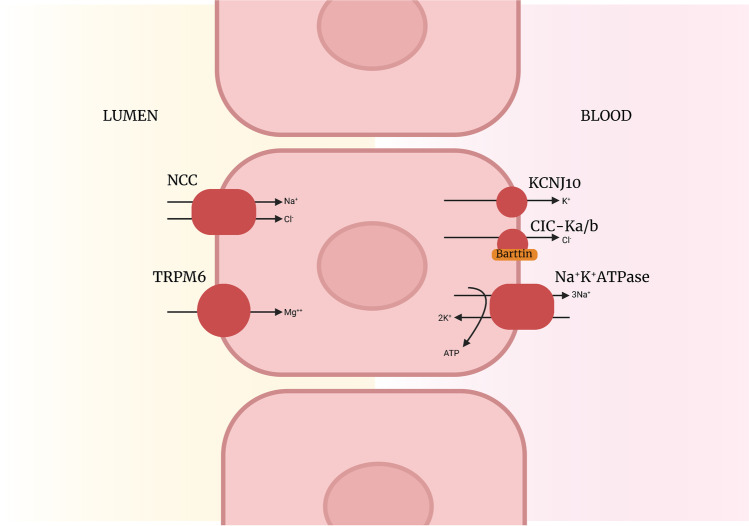
Distal convoluted tubule with the NCC implicated in Gitelman syndrome and KCNJ10 implicated in EAST syndrome. Decreased expression of TRPM6 postulated to be the cause of hypomagnesaemia in Gitelman syndrome

The mechanism of hypomagnesaemia in GS remains unclear. One theory is decreased expression of the magnesium channel TRPM6 in the DCT (Fig. 5). Hypomagnesaemia can also occur in BS (particularly Type III) due to impaired paracellular magnesium reabsorption. Hypomagnesaemia in BS is much milder than in GS [1, 28, 30, 31].

Another key differentiating feature of BS (versus GS) is hypercalciuria with nephrocalciniosis. The TAL is responsible for 20% of tubular calcium reabsorption. This occurs via paracellular mechanisms that rely on transtubular electrochemical force generated by the NKCC2 co-transport and ROMK channel (implicated in Types I and II BS, respectively) [30]. In patients with types I and II BS where this electrochemical gradient fails there is a reduction in tubular calcium reabsorption with subsequent hypercalciuria and nephrocalcinosis (see Fig. 3). Hypercalciuria occurs in types III, IV and V BS, but is less common and milder. In GS sodium reabsorption in the proximal tubule is enhanced to compensate for the downstream salt wasting. This results in increased paracellular reabsorption of calcium and subsequent hypocalciuria.

**EAST syndrome** is an AR condition secondary to variants in *KCNJ10* which encodes a rectifying potassium channel expressed in the kidney, inner ear and glial cells. It results in a tubulopathy that mimics GS with predominant extra-kidney manifestations including sensorineural deafness, ataxia, seizures and intellectual deficit [1, 3].

**HELIX syndrome** is a rare condition caused by variants in *CLDN10* which encodes proteins important for tight junction formation in the TAL and exocrine glands [24]. The net result is reduced paracellular sodium reabsorption and a salt-losing tubulopathy with differentiating features of hypermagnesemia and exocrine gland dysfunction with hypohidrosis and alacrima (see Fig. 4) [25].

More recent discoveries include variants in *RRAGD* encoding for the Rag guanosine triphosphatase that leads to a hypokalaemic, salt-losing nephropathy associated with hypomagnesaemia and dilated cardiomyopathy [26]. It also leads to a GS-like syndrome resulting from variants in mitochondrial DNA (*MT-TF*, *MT-TI*) that result in reduced NCC activity [27].

When considering hypokalaemic metabolic alkalosis with normo or hypotension, the main differentiating features between these tubular conditions are the age of onset, hypomagnesaemia and presence of hypercalciuria. Extra-kidney manifestations such as neurological involvement and exocrine gland dysfunction will guide diagnosis of rarer conditions (Table 3, Fig. 3).

### *Hypokaelaemic metabolic alkalosis with hypertension*

Hypertension in this setting reflects a state of apparent or true mineralocorticoid excess. In these conditions sodium and water are retained in lieu of potassium, resulting in a hypokalaemic metabolic alkalosis. Hypercalciuria with or without nephrocalcinosis is present in most cases due to a compensatory reduction in proximal salt reabsorption [3].

**Primary hyperaldosteronism** can be genetic or in the setting of adrenal hyperplasia or adenoma/carcinoma [23]. There are four forms of **familial hyperaldosteronism**, type 1–type 4 [32]. By the very mechanisms of these conditions, serum aldosterone is elevated, renin suppressed, and hypertension is significant.

**Liddle syndrome** is an AD condition resulting from a gain of function variant in *SCNN1A/B* which encode the alpha/beta subunits of the ENaC present in the CD [33]. Constitutive activation of this channel results in sodium and water reabsorption at the expense of potassium. This occurs independently of aldosterone and therefore aldosterone and renin are suppressed [32].

**Apparent mineralocorticoid excess** syndrome mimics primary hyperaldosteronism [23]. Causes include genetic variants leading to constitutive activity of mineralocorticoid receptors, drug toxicity or excessive licorice intake. One genetic form results from variation of *HSD11B2* that encodes for 11B-hydroxysteroid dehydrogenase involved in prevention of cortisol binding to the mineralocorticoid receptor [1, 32]. These children present with polyuria, polydipsia, failure to thrive and a hypokalaemic metabolic alkalosis with hypertension. This is again independent of aldosterone therefore renin and aldosterone are suppressed [32, 33]. Another form is Geller syndrome due to a specific variant in *NR3C2* which conveys agonism rather than antagonism of the mineralocorticoid receptor by progesterone and other steroid hormones thus resulting in early-onset hypertension that is aggravated in pregnancy [34, 35]. There are other monogenic forms of hyperaldosteronism due to excess adrenal production of mineralocorticoid related to variation in *CYP11B1* (glucocorticoid-remediable aldosteronism), *CLCN2*, *KCNJ5* and *CACNA1H* that phenocopy these intra-kidney forms of apparent mineralocorticoid excess, though manifest with extra-kidney features of hyperaldosteronism [36–39].

### Hyperkalaemic metabolic acidosis

In the CD, sodium is reabsorbed in exchange for potassium and hydrogen ions. Impaired sodium reabsorption results in reduced excretion of both hydrogen and potassium and subsequent hyperkalaemic metabolic acidosis. This state reflects an aldosterone deficiency or resistance and is referred to as a **type 4 RTA** [20, 22]***.***

Type 4 RTA has a myriad of causes including intrinsic kidney disease (CKD, obstructive uropathy), adrenal insufficiency (congenital adrenal hyperplasia), autoimmune disorders (lupus nephritis), medications (amiloride, spironolactone, calcineurin inhibitors) and genetic forms referred to as pseudohypoaldosteronism [20]. In paediatrics, type 4 RTA is most commonly observed secondary to urosepsis resulting in a reversible pseudohypoaldosteronism.

**Pseudohypoaldosteronism type 1 (PHA1)** is due to mineralocorticoid resistance. Children typically present in infancy with failure to thrive, severe hypovolaemia, hyperkalaemia and metabolic acidosis. Autosomal recessive PHA1 results from variants in the genes that encode the subunits of the EnaC channel present in the CD. Loss of function of this channel results in severe salt wasting. EnaC is also expressed in skin and lungs and therefore can lead to a cystic fibrosis-like phenotype. Autosomal dominant PHA1 results from variants in the *NR3C2* gene which encodes the mineralocorticoid receptor. Those affected by PHA1(*NR3C2)* typically present with a milder phenotype with no extra-kidney manifestations and resolution after early childhood [40]. Both conditions are the only kidney salt wasting conditions that present with hyponatraemia [1–3].

**Pseudohypoaldosteronism type 2 (PHA2) (Gordon syndrome*)*** is caused by the stimulation or prevention of degradation of the NCC co-transporter in the DCT, the same channel implicated in GS. The result is unopposed sodium reabsorption with subsequent volume expansion and hypertension. Hyperkalaemic metabolic acidosis results from suppression of sodium reabsorption in the CD leading to reduced potassium and hydrogen secretion. Variants in *WNK4*, *WNK1*, *KLHL3* and *CUL3* are implicated in PHA2 [1, 3, 20].

### Hyper- and hyponatraemia

Sodium and water homeostasis are inextricably linked. Sodium anomalies more frequently reflect volume status, and less commonly reflect a depletion of sodium stores or salt toxicity [3]. There are reviews dedicated entirely to this topic. We focus on those related to impaired tubular handling of water*,* **nephrogenic diabetes insipidus (NDI)** and **syndrome of inappropriate antidiuretic hormone (SIADH)**.

NDI results from a failure of the CD to reabsorb water in response to ADH [1, 41]. This leads to the production of dilute urine irrespective of fluid intake. In the setting of restricted access to free water (due to age) this results in volume depletion and hypernatraemia. Paired urine:serum sodium and osmolality in this setting reveal hyperosmolar serum with an inappropriately dilute urine (serum osmolality > urine osmolality) [42]. NDI most commonly results from loss-of-function variation in *AVPR2* (X-linked) with remaining cases related to *AQP2* (AD or AR) and requires distinction from central/neurohypophyseal DI due to *AVP* (AD) which is responsive to exogenous ADH (DDAVP) [43–47].

Conversely, SIADH is caused by the inappropriate reabsorption of water in the CD in response to ADH. Volume expansion and dilutional hyponatraemia follow [1, 48]. SIADH is most commonly acquired in the setting of central nervous system or respiratory pathology, an inflammatory state or post-operatively [48]. Nephrogenic syndrome of inappropriate antidiuresis (NSIAD) is a rare genetic condition that mimics SIADH [1]. It is secondary to gain-of-function variants in *AVPR2* (X-linked) leading to inappropriate water reabsorption in the absence of ADH [49, 50]. In contrast to NDI, SIADH and NSIAD present with hyponatraemia and hypo-osmolality with an inappropriately concentrated urine (serum osmolality < urine osmolality) [48].

As the *AVPR2* gene is on the X chromosome, it is males that are affected with NSIAD and in the majority of cases of NDI. Family history is important as female carriers are often partially affected and may report polydipsia and/or have a history of borderline hyponatraemia. Given sodium anomalies most commonly reflect volume status, a rigorous assessment of fluid status should always be conducted. Paired urine and serum samples assist in determining the kidney handling of salt and water.

### Hypomagnesaemia

Magnesium is a highly abundant salt in the body imperative for neuromuscular stability. Magnesium regulation is moderated by intestinal reabsorption and kidney handling and is impacted by hormonal control. In the kidney, reabsorption occurs in the proximal tubule, the TAL via paracellular mechanisms and the DCT via transcellular mechanisms [1, 3, 51].

Hypomagnesaemia is often classified according to the corresponding urinary calcium [1, 3, 51]. In the TAL, magnesium and calcium are reabsorbed by paracellular mechanisms (Fig. 4). Hypomagnesaemic conditions affecting this portion of the tubule therefore also waste calcium resulting in hypercalciuria and subsequent nephrocalcinosis. **Familial hypomagnesaemia with hypercalciuria and nephrocalcinosis** is one such condition resulting from variations in the *CLDN16/19* genes which encode claudin 16/19 respectively [52]. These transmembrane proteins facilitate paracellular magnesium and calcium reabsorption. The driving force for this process is the electrochemical gradient resulting from transtubular sodium, potassium and chloride transport (see Fig. 4). Disease-causing variations result in loss of function with subsequent salt, magnesium and calcium urinary loss.

In the DCT, hypomagnesaemia can exist in the setting of a salt-wasting syndrome or as an isolated defect affecting magnesium alone. In the former, the proximal tubule compensates for volume loss by reabsorption of salt which is paired with calcium. This leads to hypocalciuria as occurs in GS, EAST syndrome and *HNF1B-*associated tubulopathy. Conditions that affect magnesium reabsorption alone (with normocalciuria) include **familial hypomagnesaemia and hypocalcaemia**. This results from variants in *TRPM6* which encodes for the ion channel responsible for magnesium reabsorption. Hypomagnesaemia in this setting is often marked leading to reduced PTH release and subsequent hypocalcaemia.

## Management

Briefly, management of these patients requires a multidisciplinary approach (ideally in a centre of expertise) with nephrologists, general paediatricians and dieticians. Considered transition to adult nephrology models of care is encouraged in discussion with relevant services.

The mainstay of therapy is the replacement of water and electrolytes. This can present a challenge to most patients, particularly infants, who have significant fluid requirements that often compromise their ability to consume adequate calories. Early dietetic input in this setting is imperative to facilitate growth and supplemental feeds via gastrostomies are often required.

Replacement of electrolytes often requires seemingly alarming doses of potassium, sodium, bicarbonate and phosphate. Despite this, in many conditions (e.g. BS), normal serum values may not be achieved and may not be realistic therapeutic targets, instead focusing on optimising growth and avoiding symptoms.

Hypercalciuria and nephrocalcinosis are managed with adequate fluid intake and administration of citrate which binds urinary calcium and prevents crystallisation [15].

Hypertension management is disease-specific and summarised by Raina et al [32]. For example, potassium-sparing diuretics are utilised in Liddle syndrome to block EnaC.

Other disease-specific therapies are available. In BS prostaglandin inhibitors such as indomethacin and celecoxib have been utilised and result in improved growth and electrolyte profile. However, their extended use may contribute to CKD and carries a significant risk of gastric ulceration. Consensus statements on the management of BS, GS, RTA and proximal tubulopathies are readily available [14, 31, 53, 54].

## Conclusion

In summary, tubulopathies represent a complex array of conditions with non-specific presenting symptoms. The pathognomonic biochemical picture resulting from the underlying tubular defect is often the most revealing finding. It is important to identify these conditions promptly to facilitate management. The diagnosis of these conditions involves assessment of serum, urine and genetic investigation in addition to careful clinical assessment. Management requires a multi-disciplinary approach, and is generally supportive with fluid and electrolyte replacement; however, it can also be disease-specific. Finally, the discovery of genetic causes for different tubulopathies has and will continue to lead to expedited diagnoses as well as more targeted and personalised therapy and identification of at-risk relatives.

### Key summary points

- Clinically, tubulopathies can present with varied and non-descript features
- The biochemical presentation of tubulopathies is an important diagnostic tool which can guide further investigation and management
- Genetic testing in tubulopathies is important for the diagnosis and establishment of treatment-specific therapy and can facilitate ongoing counselling
- Management is multidisciplinary and focuses on the replacement of electrolytes and adequate nutrition to facilitate growth

### Multiple choice questions (answers can be found after the reference list)

1. The following is *not* a cause of a generalised proximal tubulopathy:

- Galactosaemia
- Cystinosis
- Wilson’s disease
- Proximal RTA (type 2 RTA)
- Mitochondrial ciliopathy

2. Genetic testing is important in the tubulopathies as it can

- Provide a definitive diagnosis
- Allow the commencement of disease-specific therapy
- Facilitate family planning
- All of the above

3. Which statement is *incorrect* when reviewing proximal and distal RTA?

- Both present with a hypokalaemic metabolic acidosis
- Both are associated with nephrocalcinosis
- Proximal RTA is secondary to an inability to reabsorb bicarbonate
- Distal RTA is secondary to an inability to secrete protons

4. Which statement is *incorrect* when reviewing tubulopathies that affect magnesium handling of the kidney?

- When assessing hypomagnesaemia, urinary calcium is an important tool to guide diagnosis
- Individuals with variants in *CLDN10* present with a salt wasting tubulopathy with hypomagnesaemia
- Individuals with variants in *CLDN16/19* present with a salt wasting tubulopathy with hypomagnesaemia
- Familial hypomagnesaemia with hypocalcaemia results from variants in *TRMP6*

## References

[1] Downie M, Lopez Garcia S, Kleta R, Bockenhauer D (2021). Inherited tubulopathies of the kidney: insights from genetics. Clin J Am Soc Nephrol.

[2] Kleta R, Bockenhauer D (2018). Salt-losing tubulopathies in children: what’s new, what’s controversial?. J Am Soc Nephrol.

[3] Rees L, Bockenhauer D, Webb N, Punaro M (2019). Paediatric nephrology.

[4] Klootwijk ED, Reichold M, Unwin RJ, Kleta R (2015). Renal Fanconi syndrome: taking a proximal look at the nephron. Nephrol Dial Transplant.

[5] Curthoys NP, Moe OW (2014). Proximal tubule function and response to acidosis. Clin J Am Soc Nephrol.

[6] Dantzler WH, Layton AT, Layton HE, Pannabecker TL (2014). Urine-concentrating mechanism in the inner medulla: function of the thin limbs of the loops of Henle. Clin J Am Soc Nephrol.

[7] Mount DB (2014). Thick ascending limb of the loop of Henle. Clin J Am Soc Nephrol.

[8] Subramanya AR, Ellison DH (2014). Distal convoluted tubule. Clin J Am Soc Nephrol.

[9] Roy A, Al-bataineh MM, Pastor-Soler NM (2015). Collecting duct intercalated cell function and regulation. Clin J Am Soc Nephrol.

[10] Pearce D, Soundararajan R, Trimpert C, Kashlan OB (2015). Collecting duct principal cell transport processes and their regulation. Clin J Am Soc Nephrol.

[11] Palmer BF (2015). Regulation of potassium homeostasis. Clin J Am Soc Nephrol.

[12] Klussmann E, Maric K, Rosenthal W (2000). The mechanisms of aquaporin control in the renal collecting duct. Rev Physiol Biochem Pharmacol.

[13] Gil-Peña H, Mejia N, Alvarez-Garcia O, Loredo V (2010). Longitudinal growth in chronic hypokalemic disorders. Pediatr Nephrol.

[14] Foreman JW (2019). Fanconi syndrome. Pediatr Clin N Am.

[15] Dickson FJ, Sayer JA (2020). Nephrocalcinosis: a review of monogenic causes and insights they provide into this heterogeneous condition. Int J Mol Sci.

[16] Berry MR, Robinson C, Karet Frankl FE (2013). Unexpected clinical sequelae of Gitelman syndrome: hypertension in adulthood is common and females have higher potassium requirements. Nephrol Dial Transplant.

[17] Tanudisastro HA, Holman K, Ho G, Farnsworth E (2021). Australia and New Zealand renal gene panel testing in routine clinical practice of 542 families. NPJ Genom Med.

[18] De Matteis MA, Staiano L, Emma F, Devuyst O (2017). The 5-phosphatase OCRL in Lowe syndrome and Dent disease 2. Nat Rev Nephrol.

[19] Bökenkamp A, Ludwig M (2016). The oculocerebrorenal syndrome of Lowe: an update. Pediatr Nephrol.

[20] Alexander RT, Bitzan M (2019). Renal tubular acidosis. Pediatr Clin N Am.

[21] Alexander RT, Cordat E, Chambrey R, Dimke H (2016). Acidosis and urinary calcium excretion: insights from genetic disorders. J Am Soc Nephrol.

[22] Santos F, Ordóñez FA, Claramunt-Taberner D, Gil-Peña H (2015). Clinical and laboratory approaches in the diagnosis of renal tubular acidosis. Pediatr Nephrol.

[23] Galla JH (2000). Metabolic alkalosis. J Am Soc Nephrol.

[24] Bongers E, Shelton LM, Milatz S, Verkaart S (2017). A novel hypokalemic-alkalotic salt-losing tubulopathy in patients with CLDN10 mutations. J Am Soc Nephrol.

[25] Vargas-Poussou R (2021). Pathophysiological aspects of the thick ascending limb and novel genetic defects: HELIX syndrome and transient antenatal Bartter syndrome. Pediatr Nephrol.

[26] Schlingmann KP, Jouret F, Shen K, Shen K (2021). mTOR-activating mutations in RRAGD are causative for kidney tubulopathy and cardiomyopathy. J Am Soc Nephrol.

[27] Viering D, Schlingmann KP, Hureaux M, Nijenhuis T (2021). Gitelman-like syndrome caused by pathogenic variants in mtDNA. J Am Soc Nephrol.

[28] Besouw MTP, Kleta R, Bockenhauer D (2020). Bartter and Gitelman syndromes: questions of class. Pediatr Nephrol.

[29] Walsh PR, Tse Y, Ashton E, Iancu D (2018). Clinical and diagnostic features of Bartter and Gitelman syndromes. Clin Kidney J.

[30] Blaine J, Chonchol M, Levi M (2015). Renal control of calcium, phosphate, and magnesium homeostasis. Clin J Am Soc Nephrol.

[31] Konrad M, Nijenhuis T, Ariceta G, Bertholet-Thomas A (2021). Diagnosis and management of Bartter syndrome: executive summary of the consensus and recommendations from the European Rare Kidney Disease Reference Network Working Group for Tubular Disorders. Kidney Int.

[32] Raina R, Krishnappa V, Das A, Amin H (2019). Overview of monogenic or Mendelian forms of hypertension. Front Pediatr.

[33] Kucuk N, Yavas Abalı Z, Abalı S, Canpolat N (2020). A rare cause of hypertension in childhood: answers. Pediatr Nephrol.

[34] Rafestin-Oblin ME, Souque A, Bocchi B, Pinon G (2003). The severe form of hypertension caused by the activating S810L mutation in the mineralocorticoid receptor is cortisone related. Endocrinology.

[35] Geller DS, Farhi A, Pinkerton N, Fradley M (2000). Activating mineralocorticoid receptor mutation in hypertension exacerbated by pregnancy. Science.

[36] Lifton RP, Dluhy RG, Rich GM, Cook S (1992). A chimaeric 11 beta-hydroxylase/aldosterone synthase gene causes glucocorticoid-remediable aldosteronism and human hypertension. Nature.

[37] Stowasser M, Gordon RD, Tunny TJ, Klemm SA (1992). Familial hyperaldosteronism type II: five families with a new variety of primary aldosteronism. Clin Exp Pharmacol Physiol.

[38] Geller DS, Zhang J, Wisgerhof MV, Shackleton C (2008). A novel form of human mendelian hypertension featuring nonglucocorticoid-remediable aldosteronism. J Clin Endocrinol Metab.

[39] Scholl UI, Stölting G, Nelson-Williams C, Vichot AA (2015). Recurrent gain of function mutation in calcium channel CACNA1H causes early-onset hypertension with primary aldosteronism. Elife.

[40] Geller DS, Zhang J, Zennaro MC, Vallo-Boado A (2006). Autosomal dominant pseudohypoaldosteronism type 1: mechanisms, evidence for neonatal lethality, and phenotypic expression in adults. J Am Soc Nephrol.

[41] Bockenhauer D, Bichet DG (2015). Pathophysiology, diagnosis and management of nephrogenic diabetes insipidus. Nat Rev Nephrol.

[42] Christ-Crain M, Bichet DG, Wiebke FK, Goldman MB (2019). Diabetes insipidus. Nat Rev Dis Primers.

[43] Rosen MJ, Dhawan A, Saeed SA (2015). Inflammatory bowel disease in children and adolescents. JAMA Pediatr.

[44] van den Ouweland AM, Dreesen JC, Verdijk M, Knoers NV (1992). Mutations in the vasopressin type 2 receptor gene (AVPR2) associated with nephrogenic diabetes insipidus. Nat Genet.

[45] Mulders SM, Bichet DG, Rijss JP, Kamsteeg EJ (1998). An aquaporin-2 water channel mutant which causes autosomal dominant nephrogenic diabetes insipidus is retained in the Golgi complex. J Clin Invest.

[46] Deen PM, Verdijk M, Knoers N, Wieringa B (1994). Requirement of human renal water channel aquaporin-2 for vasopressin-dependent concentration of urine. Science.

[47] Wahlstrom JT, Fowler MJ, Nicholson WE, Kovacs WJ (2004). A novel mutation in the preprovasopressin gene identified in a kindred with autosomal dominant neurohypophyseal diabetes insipidus. J Clin Endocrinol Metab.

[48] Moritz ML (2019). Syndrome of inappropriate antidiuresis. Pediatr Clin N Am.

[49] Erdelyi LS, Mann WA, Morris-Rosendahl DJ, Grob U (2015). Mutation in the V2 vasopressin receptor gene, AVPR2, causes nephrogenic syndrome of inappropriate diuresis. Kidney Int.

[50] Feldman BJ, Rosenthal SM, Vargas GA, Fenwick RG (2005). Nephrogenic syndrome of inappropriate antidiuresis. N Engl J Med.

[51] Viering D, De Baaij J, Walsh SB, Kleta R (2017). Genetic causes of hypomagnesemia, a clinical overview. Pediatr Nephrol.

[52] Vall-Palomar M, Madariaga L, Ariceta G (2021). Familial hypomagnesemia with hypercalciuria and nephrocalcinosis. Pediatr Nephrol.

[53] Trepiccione F, Walsh SB, Ariceta G, Boyer O (2021). Distal renal tubular acidosis: ERKNet/ESPN clinical practice points. Nephrol Dial Transplant.

[54] Blanchard A, Bockenhauer D, Bolignano D, Calo LA (2017). Gitelman syndrome: consensus and guidance from a Kidney Disease: Improving Global Outcomes (KDIGO) Controversies Conference. Kidney Int.

[55] Payne RB (1998). Renal tubular reabsorption of phosphate (TmP/GFR): indications and interpretation. Ann Clin Biochem.

[56] Elisaf M, Panteli K, Theodorou J, Siamopoulos KC (1997). Fractional excretion of magnesium in normal subjects and in patients with hypomagnesemia. Magnes Res.

